# Meta-Analysis of the Relationship between Deep Brain Stimulation in Patients with Parkinson's Disease and Performance in Evaluation Tests for Executive Brain Functions

**DOI:** 10.1155/2017/9641392

**Published:** 2017-02-08

**Authors:** A. M. Martínez-Martínez, O. M. Aguilar, C. A. Acevedo-Triana

**Affiliations:** ^1^Department of Psychology, Pontificia Universidad Javeriana, Bogotá, Colombia; ^2^Department of Brain Repair and Rehabilitation, University College London, London, UK

## Abstract

Parkinson's disease (PD) is a neurodegenerative condition, which compromises the motor functions and causes the alteration of some executive brain functions. The presence of changes in cognitive symptoms in PD could be due to the procedure of deep brain stimulation (DBS). We searched in several databases for studies that compared performance in executive function tests before and after the DBS procedure in PE and then performed a meta-analysis. After the initial search, there were 15 articles that specifically evaluated the functions of verbal fluency, working memory, cognitive flexibility, abstract thinking, and inhibition. It was found that there were differences in the evaluation of the cognitive functions in terms of the protocols, which generated heterogeneity in the results of the meta-analysis. Likewise, a tendency to diminish functions like verbal fluency and inhibition was found, being this consistent with similar studies. In the other functions evaluated, no difference was found between pre- and postsurgery scores. Monitoring of this type of function is recommended after the procedure.

## 1. Introduction

Parkinson's disease (PD) is a common, progressive and incurable neurodegenerative disease with an unknown etiology, whose main symptoms include motor alterations such as shaking, an abnormal increase in muscle tone, bradykinesia, postural instability, impaired balance and walking, and emotional inexpressiveness [1–6]. In postmortem studies of patients with PD, these clinical features have been directly related to the reduction of dopamine neurons in the cortical-thalamus-striated loop [1, 4–7], mitochondrial alterations [4], and the presence of clusters of *α*-synuclein presynaptic protein, known as Lewy bodies [4, 7, 8].

From a neurological perspective, the symptoms of PD have been considered to be the result of alterations in the communication between the direct/indirect motor control pathways of the basal ganglia. According to this “classic” model, this deficiency in communication is given by a reduction in the dopaminergic transmission which in turn results in the diminished inhibition of the indirect pathway, the excitation of the direct pathway, and the excessive activation in the discharge of internal globus pallidus (GPi) and an inhibition of the thalamic cortical motor system [9, 10]. Given the model's limitations in explaining PD systems other than the motor ones, it is recognized that the Cortico-Basal Ganglia-Thalamus loop is implied in eye movement control functions (the oculomotor circuit) [11], memory and spatial orientation (dorsolateral prefrontal circuit) [10], behavioral adjustment and control, and the reward and punishment system (lateral orbitofrontal circuit) [9].

It has been suggested that cognitive [9], emotional [12], and behavioral [13] alterations can be generated in the BG-cortex communication. In this same sense, although it has not been a characteristic present in all the reports, a significant metabolic reduction has been found in patients with Parkinson's disease, predominantly in areas of parietal and medial frontal association [5].

Among the nonmotor clinical symptoms there is a broad spectrum of alterations at cognitive [1, 9, 14], emotional, mood [15], behavioral [16, 17], and psychiatric levels [17, 18]. In some cases, the cognitive deficit is comparable to executive alterations similar to patients with lesions in the frontal lobe, given the reduction of dopaminergic activity in the frontostriatal circuits, but without being considered a “frontal lobe syndrome,” leading to episodic alterations and visuospatial and verbal fluency dysfunctions [9, 19]. Previous studies have reported on the appearance of alterations in tasks that assess executive brain functions, such as verbal fluency [20], Trail Making Test (TMT-B), Wisconsin Card Sorting Test (WCST), Stroop [19], Theory of Mind [21, 22], and timing deficits [23].

The treatments reported for PD include dopamine antagonist pharmacological treatments [2, 3, 24], physical therapy [25, 26], genetic therapy [24], transcranial magnetic stimulation [15, 27, 28], injury to the subthalamic nucleus [29], and high frequency deep brain stimulation (DBS) [30–37]. The latter has been proven to reduce the severity of motor symptoms, to reduce pharmacological treatment significantly, and to improve patients' quality of life [1, 31, 32, 35, 36, 38–40]. DBS has been reported in subcortical structures such as the subthalamic nucleus (STN), the internal globus pallidus (GPi), the pedunculopontine nucleus (PPN), and prelemniscal radiation [35, 36, 41–45]. Stimulator frequency depends on the patient's clinical aspects and the location of the electrodes [31, 42].

In the assessment of nonmotor symptoms (disturbed sleep patterns, salivation, mood, cognitive, and executive function), it has been reported that the DBS procedure fosters a number of changes. In DBS of the STN, Bickel et al. [29] found that general performance remained constant in frontal executive function tests [16, 23]. In bilateral DBS of the STN, significant improvement has been reported in the learning of verbal information and visuoconstructive skills when there is increased stimulator amplitude [38, 46]. Inasmuch as the DBS of the PPN, improvements have been reported in terms of tasks related to working memory (MT) [23, 47]. It has also been reported that STN-DBS is involved in the generation of impulse control disorders but that this is not a maintained effect [48].

Some studies have identified metabolic changes associated with execution of tasks, reporting that there is an activity reduction network in PD that includes the supplementary motor area (preSMA), precuneus, the inferior parietal lobe, and the left prefrontal cortex, as well as an increase in the cerebellar vermis and the dentate nucleus, probably due to the cerebellum-BG connections [5, 49]. Changes in the structures of this area can be seen in tasks that involve cognitive performance which may suggest that alterations in the network play a role in other cognitive functions [50].

A central aspect of this study is the DBS procedure and its impact on nonmotor symptoms in PD [40]. Thus, a meta-analysis of 28 studies was carried out of studies by Parsons et al. [51]. The authors analyzed the cognitive consequences of STN-DBS, concluding that the procedure presents a small effect on all the cognitive domains assessed, except on verbal fluency, shedding light on a lower statistically significant performance in phonetic and semantic verbal fluency tests after DBS.

Given the lack of consensus inasmuch as the impact of the DBS procedure on executive brain functions specifically, the aim of this study was to identify changes in the executive brain functions tests after DBS in six months or more, reported in the last ten years. To do this, we used studies that showed results for before and after DBS and analyzed these using meta-analysis.

## 2. Method

### 2.1. Study Selection

An information search was carried out in the Scopus databases using the following key words: “deep AND brain AND stimulation AND Parkinson AND executive AND functions.” The search yielded 126 articles that covered the 2005–2015 period. Using the same key words, the Pubmed database yielded 39 results; the Web of Science (WOS) database, 104 results; the Sage journals database, 142 results; the Taylor Francis Online database, 125 results; the Wiley Online Library, 1362 results; the Embase database, 149 results; and Proquest, 3295 results. Finally, using the PsychNET database, the search initially gave no results; thus it was modified using the words “Parkinson AND DBS,” yielding 6 results. This gave a total of 5348 records in 9 databases. The results were subsequently grouped by year and types of journal articles.

**Figure 1:**
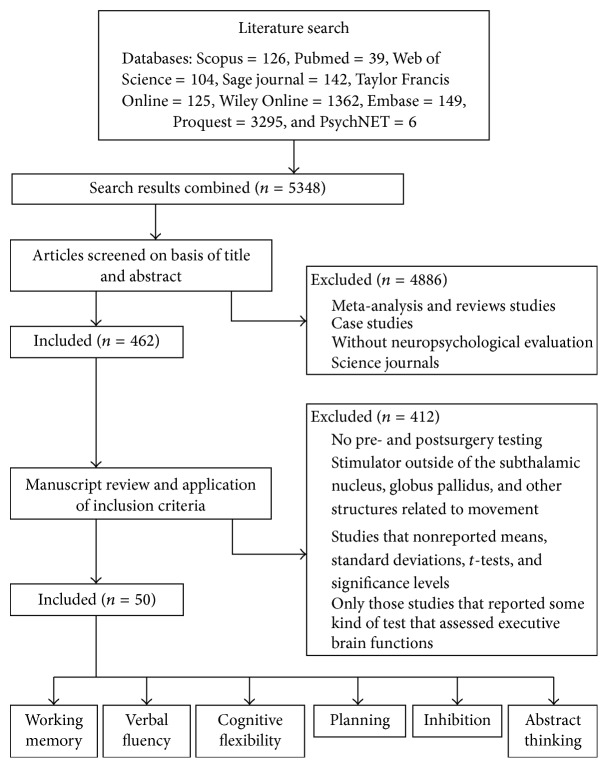
Flow diagram of study selection. Adapted from Liberati et al. [53].

The cleaning process was undertaken in two phases. The first was a selection of articles published in science journals, excluding reviews, meta-analyses, and case studies. The results for this first phase are shown in Figure 1.

### 2.2. Study Inclusion Criteria

The studies were selected considering the following recommendations: (a) types of design; (b) types of intervention; (c) participant characteristics; (d) statistical data; and (e) the tests used [52]. All the reported studies were written in English and dated between 2005 and 2015. The inclusion criteria for this meta-analysis were the following: (a) pre- and postsurgery testing of stimulator implantation; (b) for the target, the subthalamic nucleus, globus pallidus, and other structures related to movement; (c) sociodemographic variables were not taken into account for participant characteristics (age, how long the patient has had the disease, educational level, and type of medication); (d) studies that reported means, standard deviations, *t*-tests, significance levels; and (e) only those studies that reported some kind of test that assessed executive brain functions (working memory, verbal fluency, cognitive flexibility, planning, inhibition, and abstract thinking) and processing speed. Figure 1 outlines the search procedure. Nonadditional studies were identified by contacting clinical experts and searching bibliographies in local repositories.

### 2.3. Codification of the Studies

The studies were codified independently by 4 researchers and the codified information was subsequently corroborated. The following characteristics were taken into account for the codification: (a) identification of the study by the first author's surname and the year of publication; (b) the number of participants; (c) the study design (before and after surgery; only after surgery; cases and controls; and correlational); (d) location of implanted deep brain stimulation (subthalamus; globus pallidus; and other); (e) parameter related to the stimulator (pulse, frequency, voltage, and electrode type); (f) schooling (secondary education, university education, graduate studies, none, and not reported); (g) age (under 50, 51–60, 61–70, over 70, and not reported); (h) time of suffering from PD symptoms before brain stimulation surgery (short, less than 5 years; medium, 6–10 years; late, more than 10 years; and not reported); (i) sex (men, women, mixed, and not reported); (j) socioeconomic status (reported, not reported); (k) type of medication; (l) results values associated with the executive brain functions tests undertaken (Table 1); and (m) time before assessment after the stimulator implantation surgery. When the information was codified for the meta-analysis, the time after stimulator implantation variable was not taken as a homogenization criterion for the studies. That is, for those that presented more than one posterior measurement, the measurement closest to 12 months after the surgery was used.

The executive brain functions considered in the study analysis include verbal fluency, cognitive flexibility, working memory, processing speed, behavioral inhibition, and planning (Table 2). Following Parsons et al. [51], the verbal fluency assessment tasks were separated due to the reported systematic reduction of the verbal fluency function in patients with PD with DBS and the difference (category or letters) in terms of task processing.

### 2.4. Statistical Analysis

The mean scores of the tests undertaken were calculated and Hedges's *g* values and standard error (SE) for each study are reported together with 95% confidence intervals (CIs). It was assumed that if value *I*^2^ was below 50% of heterogeneity, a meta-analysis with a fixed effects model would be applied; otherwise, a random effects model would be used [57].

To assess the publication bias, a funnel plot was used for each of the meta-analyses [58]. The meta-analysis and funnel plot were carried out using the Comprehensive Meta-analysis 2.0 software. *p* < 0.05 value was considered to have statistical significance.

## 3. Results

Once the search was refined, 5348 studies were analyzed (Figure 1). Figure 1 shows the results of the initial search.

### 3.1. Descriptive

The descriptive results are shown in Table 2 which outlines the studies, number of patients, age, time of illness, schooling, PD alteration scores, and other values reported for the studies.

### 3.2. Meta-Analysis

For this study, a fixed effects model was used due to two conditions. First, the conditions of the participants and characteristics of the disease are similar among the studies and with this a population effect size is theoretically assumed [52, 59]. On the other hand, given that it was previously assumed that the percentage of heterogeneity exceeded 50% measured by coefficient *I*^2^, a random effects model was used [57]. It is important to signal that only one study has results of GPi stimulation (Rothlind, 2015) and because of this the results and figures were not separate.

### 3.3. Verbal Fluency

**Figure 2:**
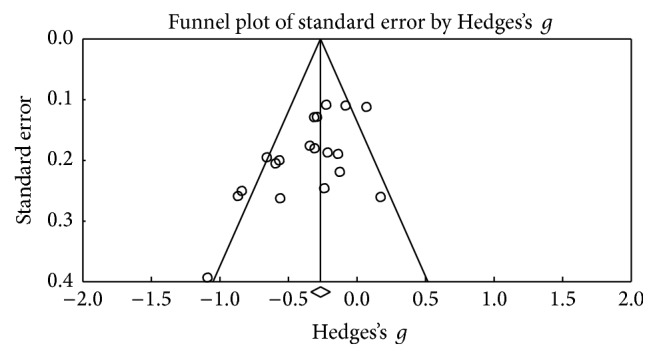
Funnel plot for standard error in publications of verbal fluency.

**Figure 3:**
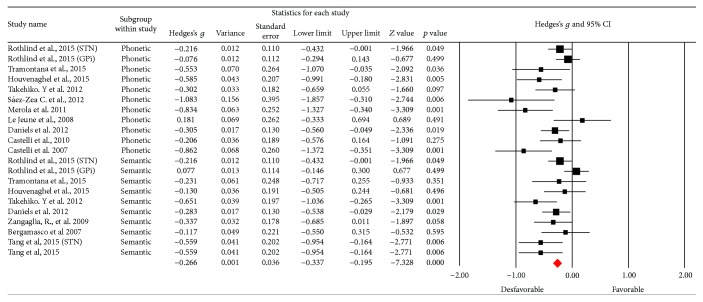
Meta-analysis of verbal fluency comparing before and after DBS surgery. Verbal fluency was separated in phonetic and semantic parts. STN = subthalamic nucleus; GPi = internal globus pallidus.

Figure 2 outlines the funnel plot of the SE for studies of verbal fluency and there is no bias in the studies reported [58]. In this category, we obtained 21 studies that were clustered depending on the evaluation modality (semantic or phonetic), Hedges's *g* was used to determine the size of the effect, obtaining a medium effect size (Hedges's *g* = −0.266; SE = 0.036; CI −0.337 to −0.195), which showed heterogeneity (*Q*_(20)_ = 42,911; *p* = 0.002) within an average percentage (*I*^2^ = 53,39%), which, when in excess of 50%, led to the application of a random model [60]. The results also showed a significant reduction in performance in the test after the DBS procedure (*Z* value = −5,607; *p* < 0.001) (Figure 3).

### 3.4. Cognitive Flexibility

**Figure 4:**
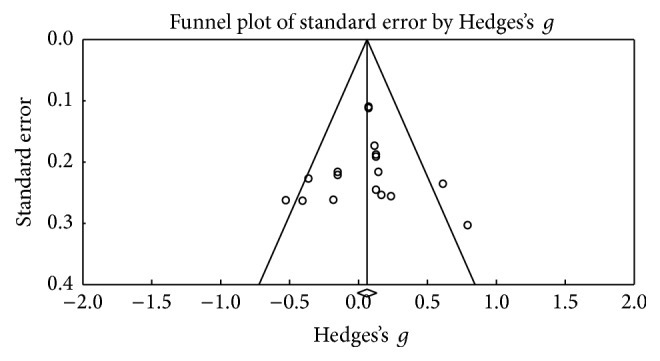
Funnel plot for standard error in publications of cognitive flexibility (WSCT).

**Figure 5:**
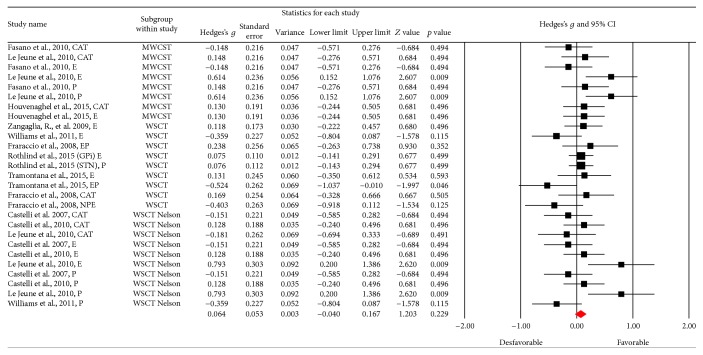
Meta-analysis of WSCT comparing before and after DBS surgery. The Wisconsin Short Card Test had three versions. Version one: MWCST = modified WCST; version two: WSCT; and version three: WSCT Nelson version.

This function was assessed based on the Wisconsin Shorting Card Test (WSCT) and Trail Making Test (TMT) in its B and B-A versions.  Figure 4 shows the funnel plot used for the SE in WSCT; the figure shows three points outside the projection in the upper threshold, but these are shown as equivalents to the points on the lower threshold. The meta-analysis obtained 27 results in which the Wisconsin Shorting Card Test (WSCT) in its different versions (Nelson or Modified) was assessed, bearing in mind the different types of scores (errors, perseverations, or categories). A small effect size was found (Hedges's *g* = 0.064; SE* = *0.053; CI* −*0.04 to 0.167), showing heterogeneity (*Q*_(26)_ = 44,94; *p* = 0.012) within an average percentage (*I*^2^ = 42,14%), but without exceeding 50% [43, 60]. There seems to be no significant change in the test scores after the DBS procedure (*Z* value = 1,656; *p* = 0.098) (Figure 5).

**Figure 6:**
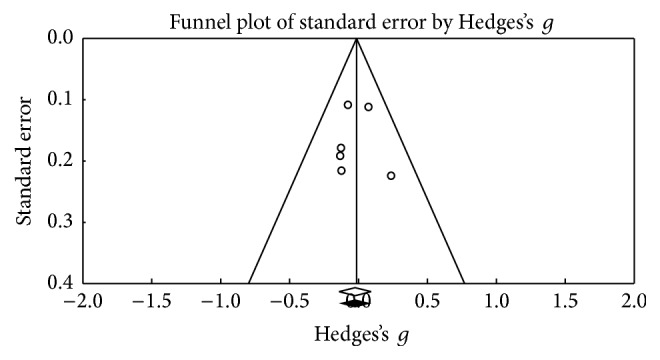
Funnel plot for standard error in publications of Trail Making Test (TMT-A).

**Figure 7:**
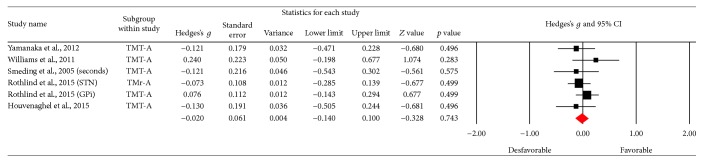
Meta-analysis of TMT-A comparing before and after DBS surgery.

**Figure 8:**
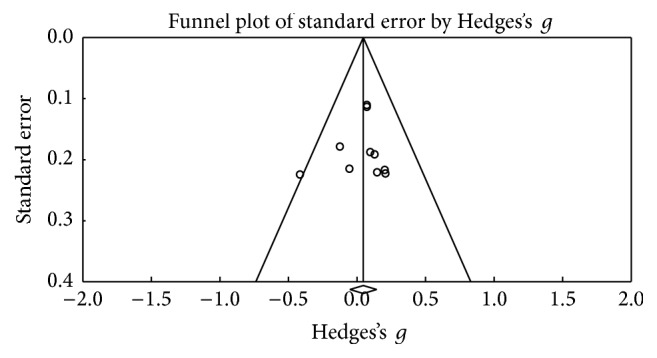
Funnel plot for standard error in publications of Trail Making Test (TMT-B).

**Figure 9:**
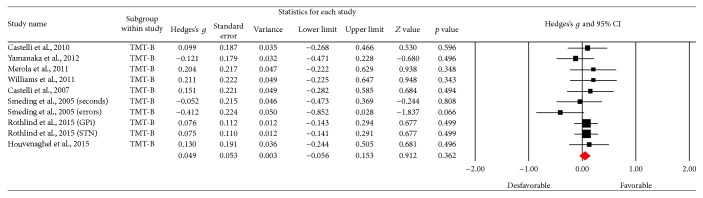
Meta-analysis of TMT-B comparing before and after DBS surgery.

**Figure 10:**
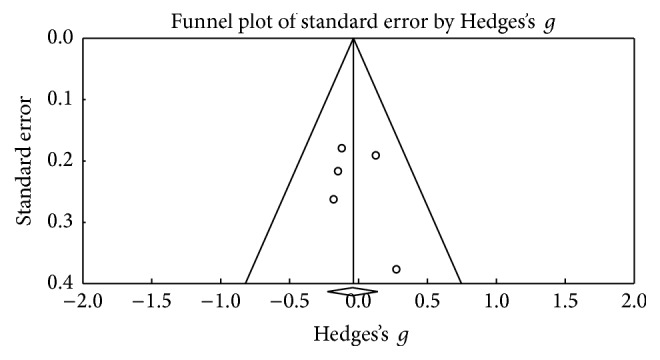
Funnel plot for standard error in publications of Trail Making Test (TMT-AB).

**Figure 11:**
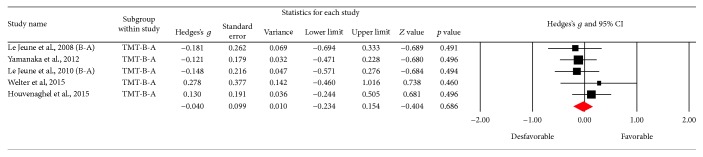
Meta-analysis of TMT-AB comparing before and after DBS surgery.

Using the Trail Making Test (TMT-A), 6 results were obtained; Figure 6 shows the funnel plot for the SE of the test, and no biases are observed. The studies in the meta-analysis reveal no differences in terms of execution (*Z* value = −0.328; *p* = 0.743), the effect detected was small (Hedges's *g* = −0.02; SE = 0.061; CI −0.14 to 0.1), and the results showed homogeneity (*Q*_(5)_ = 3,202; *p* = 0.669) within the 0% value (*I*^2^ = 0%) (Figure 7). With respect to the other tests for the same function such as version B of the TMT, 10 of the results found did not reveal an important change between the applications (*Z* value = 0.912; *p* = 0.362), the effect detected was small (Hedges's *g* = −0.02; SE* = *0.053; CI* −*0.056 to 0.153), and the results showed homogeneity (*Q*_(9)_ = 6,973; *p* = 0.64) at a very low percentage (*I*^2^ = 0%) (Figure 9).  Figure 8 presents the funnel plot for the SE of the TMT-B. Finally, for the TMT-B-A version (5 results) the funnel plot is presented in Figure 10 and no differences were found between applications before and after the DBS procedure (*Z* value = −0.404; *p* = 0.686). The effect detected was small (Hedges's *g* = −0.04; SE* = *0.099; CI* −*0.234 to 0.154), and the results showed homogeneity (*Q*_(4)_ = 2,251; *p* = 0.69) at a very low percentage (*I*^2^ = 0%) (Figure 11).

### 3.5. Abstract Thinking

**Figure 12:**
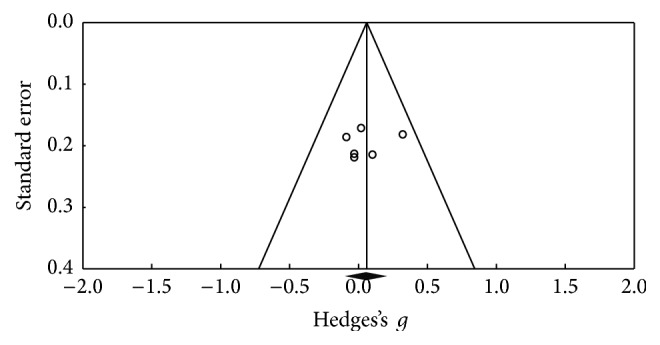
Funnel plot for standard error in publications of Raven Matrix.

**Figure 13:**
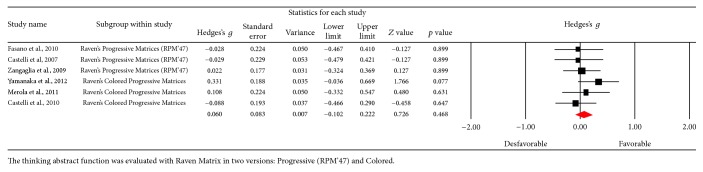
Meta-analysis of Raven Matrix comparing before and after DBS surgery.

Figure 12 shows the funnel plot and no bias among the studies was observed. In this category, 6 studies were obtained, and no changes in test performance were observed after the DBS procedure (*Z* value = 0.722; *p* = 0.471) (Figure 13). A small effect size was obtained (Hedges's *g* = 0.058; SE = 0.080; CI −0.099 to 0.215), and the result showed homogeneity (*Q*_(5)_ = 3,088; *p* = 0.686) within a low percentage (*I*^2^ = 0%).

### 3.6. Working Memory

**Figure 14:**
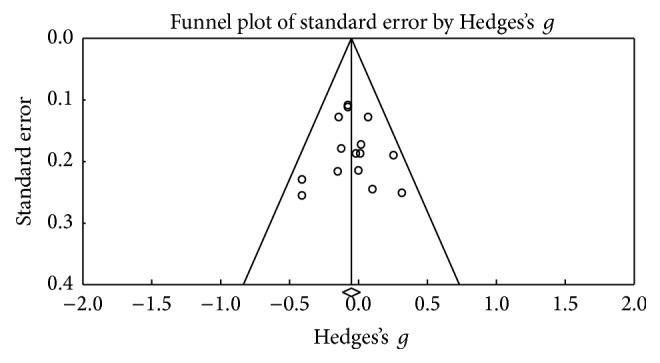
Funnel plot for standard error in publications of Digit Span Test (DST).

**Figure 15:**
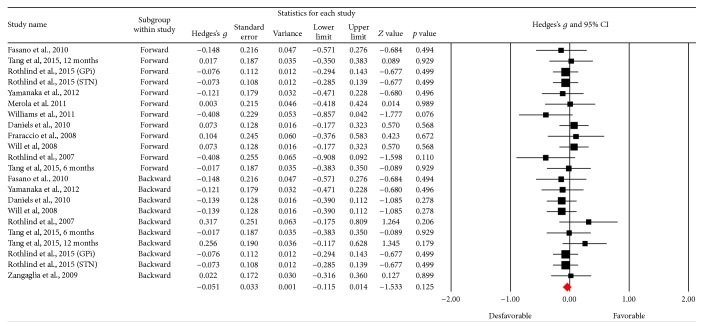
Meta-analysis of DST comparing before and after DBS surgery.

Figure 14 shows the funnel plot and no bias among the studies is observed. In this category, 22 results were obtained, and no changes in test performance were observed after the DBS procedure (*Z* value = −1,533; *p* = 0.125) (Figure 15). A small effect size was obtained (Hedges's *g* = −0.051; SE* = *0.033; CI* −*0.115 to 0.014), and the result showed homogeneity (*Q*_(21)_ = 13,682; *p* = 0.883) at a low percentage (*I*^2^ = 0%).

### 3.7. Inhibition

**Figure 16:**
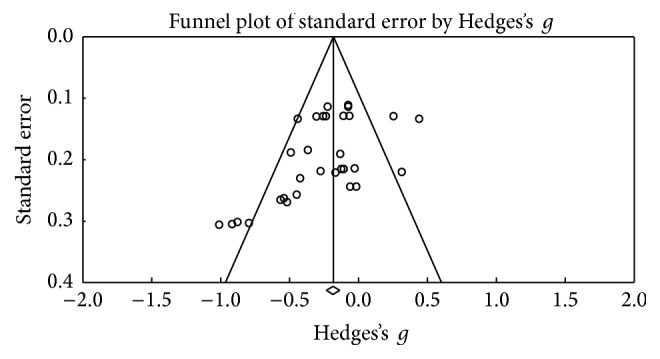
Funnel plot for standard error in publications of Stroop Test.

Figure 16 shows the funnel plot for inhibition; a number of scores outside the lower and upper thresholds were obtained suggesting a bias in the studies. However, when visual criteria were applied, the bias does not present itself fully, and there are a number of points close to the upper threshold. What does result from this analysis is a high degree of heterogeneity between the studies (*Q*_(40)_ = 88,95; *p* < 0.001) corresponding to over 89% of the variability among them (*I*^2^ = 55,03%). In this category, 41 results were obtained.

**Figure 17:**
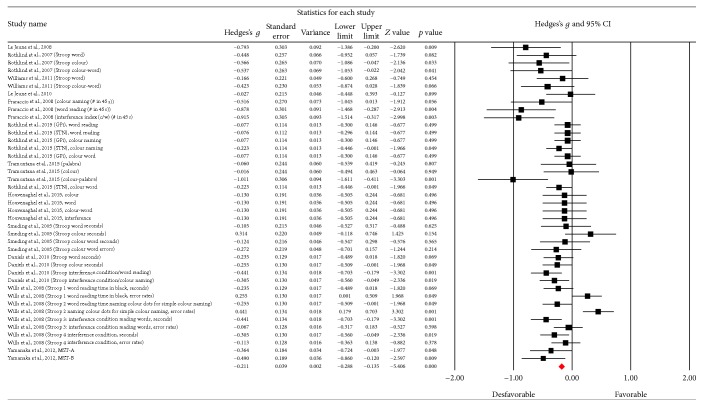
Meta-analysis of Stroop Test comparing before and after DBS surgery.

Given this heterogeneity, a random model meta-analysis was applied and a change in the execution of the test was observed as it significantly reduced after the DBS procedure (*Z* value = −0.406; *p* < 0.001) (Figure 17). A small effect size was found (Hedges's *g* = −0.211; SE = 0.039; CI −0.268 to −0.135).

## 4. Discussion

The results of this study were found to correspond to similar studies in which there is a general reduction of executive brain functions after the DBS procedure. This does not seem to have an impact on quality of life given the improvement of motor symptoms [19, 51, 61]. It is worth highlighting that the study of EF has shown a reduction in tasks such as WCST, verbal fluency, and Stroop in patients with PD before the DBS procedure. This could be explained by alterations in the BG-dorsolateral prefrontal cortex loop in relation to the reduction of dopamine in the nigrostriatal and mesocortical pathways [10].

In general, the study of EF presents a difficulty in terms of the unification of concepts. It has been recognized that the lack of unity in the measurements and significance makes it difficult to establish the relationship with clinical aspects and to explain the improvement or reduction of the functions tested [19]. Following Kudlicka et al. [19], the conclusions are due to the performance in the tests presented without this being an exhaustive analysis of EF. With this, it was found in a number of studies that the same test was used to assess various functions. The lack of representation of Latin American individuals and the lack of studies carried out in Latin America are notable.

The meta-analysis studies and systematic reviews have identified important aspects of PD that could explain part of the emotional functioning, that is, a deficit of emotional recognition which, although not reported in other clinical studies of PD, could help improve communication processes and mood alterations [62]. Such studies can also help us understand the possible relationship between structures such as STN and the structures involved in emotional and cognitive processes [55] and, as such, better understand the disease as a whole.

In the case of the verbal fluency tests, a deterioration has been reported for PD both with pharmacological treatment and with DBS [54]. There is a change in verbal fluency performance with DBS, and this is coherent with other studies and meta-analyses in which a reduction in performance is reported [46, 51, 56]. This alteration has been related to the position of the electrodes on the STN in the left hemisphere [63]. In neuroimaging studies of patients with PD, an associative-type reduction of the metabolic function of the frontal and parietal areas has been found [5], and other studies suggest that the striate nucleus may play a dissociable role in motor control and language cognitive processes, which would mean that different patterns of stimulation would affect the structures of the basal ganglia and cortical regions in different ways. This, in turn, explains why some patients improve in terms of their language articulation and at the same time present a reduction in their verbal fluency after DBS [51]. It has also been reported that the stimulation may cause a decrease of activity in the temporal cortex and inferior frontal areas in the left hemisphere, which would decrease verbal fluency, especially of the phonological kind [64]. Nevertheless, it is necessary to highlight that these hypotheses are still under study.

Inasmuch as heterogeneity, this can be explained based on the variability in the rigorousness of the application and the standardized test to assess it. Given that the reported heterogeneity is close to 45%, it is proposed that the effect detected cannot necessarily be attributed to the DBS procedure.

Inasmuch as cognitive flexibility, the tests assessed do not show a significant change, despite being one of the functions which in other studies is reported as favorable [56]. Similarly, the working memory function has been proposed as one of the aspects that becomes altered in PD. More alterations have been identified in the visuospatial modality than the verbal modality [47, 65], and no significant changes are reported in this study for after DBS.

Inasmuch as the Stroop, no clear effect was identified perhaps due to the high heterogeneity of the studies that may be assumed as being derived from the alternative forms of the test [56].

On the other hand, another type of meta-analysis in PD has been carried out, linking the disease to different levels; for example, a genetic level which shows susceptibility to PD depending on polymorphisms in monoamine oxidase genes (MAO) [66], with other diseases or effects of the transcranial magnetic stimulation [15, 27]. This sheds light on the fact that there is a variety of studies that attempt to explain specific aspects of PD, but, as yet, with no unity of analysis that allows us to understand the diversity of the symptoms of patients with PD.

One of the difficulties reported in establishing a STN-DBS effect in systematic changes in the patients and that explains the variability of the effects, as well as the tasks, is the exact location of the electrodes. In this respect, it has been found that although the procedure is carried out in STN, the area of location, the area of active stimulation, or the volume of electrode contact is not always homogeneous [6, 63, 67].

Another of the major difficulties in the systematic assessment of the changes realized by the DBS procedure is the lack of standardized tests to measure the functions [16]. In this study, high variability was found in the versions of some of the tests which could be a factor that contributes to the heterogeneity. On the other hand, it has also been proposed that the alterations presented in PD do not always correlate with the specific alterations related to the treatment (e.g., pharmacological). Thus, the alterations in the different domains and the lack of EF improvement after DBS treatment may respond to a nonlinear model that involves different and complex circuits that are not necessarily modified by STN-DBS [68].

Finally, one of the important limitations to detecting of the effects of the procedure is the lack of control or placebo groups that would allow the identification of DBS [56].

## Figures and Tables

**Table 1:** Demographic and clinical aspects of patients in studies and frequency of neuropscyhological tests.

Study	*N*	Country	Design	Target	Age	Education (years)	Onset Parkinson disease	Hoehn & Yahr stage	Time to postevaluation in months	Pulse	Frequency	Voltage	Electrodes
Verb fluency-semantic													
Takehiko-Yamanaka et al. (2012)	30	Japan	Pre-post	STN	61,1 (9,1)	12,5 (4,5)	11,5 (5,7)	—	1, 12	90 *μ*s	130 Hz	2,4	Monopolar
Daniels (2012)	60	Germany	Pre-post + control	STN	60,2 (7,9)	—	13,8 (6,3)	2,29 (0,72)	6	60 *μ*s	130 Hz	—	—
Castelli et al. [54]	19	Italy	Pre-post	STN	62,1 (4,2)	—	14,7 (5)	—	17	Right 61,6 (6,9), left 61,6 (6,9)		Right 3,2 (0,4), left 3,2 (0,5)	Monopolar bilateral
Zangaglia et al. (2009)	65	Italy	Pre-post + control	STN	58,84 (7,70)	7,31 (3,21)	11,84 (5,07)	2,34 (0,43)	1, 6, 12, 24, 36				Semimicroelectrode
Tang (2015)	27	China	Pre-post	STN					6, 12	—	—	—	Bilateral
Rothlind (2015)	164	USA	Pre-post + control	STN + GPi	62,3 (8,9)	14,8 (3)	12,8 (5,5)	3,3 (0,9)	6	—	—	—	Bilateral
Houvenaghel (2015)	26	France	Pre-post + control	STN	55,8 (6,2)	10,1 (2,4)	11,7 (4)	1,8 (0,8)	3	—	—	—	Bilateral
Tramontana (2015)	30	USA	Pre-post + control	STN + medicine	60 (7)	—	2,2 (1,4)	2	6 y 12	—	—	—	Bilateral

Verb fluency-phonemic													
Takehiko-Yamanaka (2012)	30	Japan	Pre-post	STN	61,1 (9,1)	12,5 (4,5)	11,5 (5,7)	—	1, 12	90 *μ*s	130 Hz	2,4 V	Monopolar
Merola et al., (2011)	20	Italy	Pre-post + control	STN + OTHER	66,5 (2,5)	—	16,4 (4,3)	—	14	—	—	—	—
Daniels et al., 2010	60	Germany	Pre-post + control	STN	60,2 (7,9)	—	13,8 (6,3)	2,29 (0,72)	6	60 *μ*s	130 Hz	—	—
Castelli et al., [54]	19	Italy	Pre-post	STN	62,1 (4,2)	—	14,7 (5)	—	17	Right 61,6 (6,9), left 61,6 (6,9)		Right 3,2 (0,4), left 3,2 (0,5)	Monopolar bilateral
Le Jeune et al. [55]	13	France	Pre-post + control	STN	57 (7,8)	—	10,9 (2,2)	—	—	64,6	R 135,3 Hz, L 136,5 Hz	Right 2,3 V y, left 2,4 V	Quadripolar
Sáez-Zea, et al. (2012)	9	Spain	Pre-post	STN	54 (14)	—	12 (2)	3		—	—	—	—
Castelli et al., (2010)	27	Italy	Pre-post	STN	60,6 (6,7)	8 (4,1)	15,3 (5,1)	—	1	—	—	—	—
Rothlind (2015)	164	USA	Pre-post + control	STN + GPi	62,3 (8,9)	14,8 (3)	12,8 (5,5)	3,3 (0,9)	6	—	—	—	Bilateral
Houvenaghel (2015)	26	France	Pre-post + control	STN	55,8 (6,2)	10,1 (2,4)	11,7 (4)	1,8 (0,8)	3	—	—	—	Bilateral
Tramontana (2015)	30	USA	Pre-post + control	STN + medicine	60 (7)	—	2,2 (1,4)	2	6 y 12	—	—	—	Bilateral

Wisconsin Card Sorting Test (WCST)													
Zangaglia (2009)	32	Italy	Pre-post + control	STN	58,84 (7,70)	7,31 (3,21)	11,84 (5,07)	2,34 (0,43)	1, 6, 12, 24, 36				Semimicroelectrode
Fraraccio, (2008)	15	Canada	On-off	STN	58,1 (7,46)	11,3 (3,97)	13,6 (4,39)	—	19	Left: 94,0, right: 94,0	Left: 185 Hz, right: 185 Hz	Left: 2,8, right: 2,8	Quadripolar
Williams et al. (2011)	19	USA	Post	STN	62,1 (10,3)	13,6 (1,71)	10,1 (6,24)	1,5–3,0	24	—	—	—	—
Rothlind (2015)	164	USA	Pre-post + control	STN + GPi	62,3 (8,9)	14,8 (3)	12,8 (5,5)	3,3 (0,9)	6	—	—	—	Bilateral
Tramontana (2015)	30	USA	Pre-post + control	STN + medicine	60 (7)	—	2,2 (1,4)	2	6 y 12	—	—	—	Bilateral

Nelson Modified WSCT													
Castelli et al. [54]	19	Italy	Pre-post	STN	62,1 (4,2)	—	14,7 (5)	—	17	Right 61,6 (6,9), left 61,6 (6,9)		right 3,2 (0,4), left 3,2 (0,5)	Monopolar bilateral
Castelli, (2010)	27	Italy	Pre-post	STN	60,6 (6,7)	8 (4,1)	15,3 (5,1)	—	1	—	—	—	—
Fasano (2010)	20	Italy	Pre-post	STN	56,9 (7,2)	—	13,7 (4,8)	3	5, 8	60 *μ*s	130 Hz	—	—
Le juene, (2010)	13	France	Pre-post	STN	57 (7,8)	—	10,9 (2,2)	—	3	2,7 (±0,5)	68,7 (±13,9)	38,1 (±17,1)	Quadripolar
Le Jeune et al., [55]	13	France	Pre-post + control	STN	57 (7,8)	—	10,9 (2,2)	—	—	64,6	R 135,3 Hz, L 136,5 Hz	Right 2,3 V, left 2,4 V	Quadripolar
Houvenaghel (2015)	26	France	Pre-post + control	STN	55,8 (6,2)	10,1 (2,4)	11,7 (4)	1,8 (0,8)	3	—	—	—	Bilateral

Trail Making Test (TMT-B)													
Takehiko-Yamanaka (2012)	30	Japan	Pre-post	STN	61,1 (9,1)	12,5 (4,5)	11,5 (5,7)	—	1, 12	90 *μ*s	130 Hz	2,4 V	Monopolar
Merola (2011)	20	Italy	Pre-post + control	STN + OTHER	66,5 (2,5)	—	16,4 (4,3)	—	14	—	—	—	—
Williams (2011)	19	USA	Post	STN	62,1 (10,3)	13,6 (1,71)	10,1 (6,24)	1,5–3,0	24	—	—	—	—
Castelli et al. [54]	19	Italy	Pre-post	STN	62,1 (4,2)	—	14,7 (5)	—	17	Right 61,6 (6,9), left 61,6 (6,9)		Right 3,2 (0,4), left 3,2 (0,5)	Monopolar bilateral
Le juene, (2010)	13	France	Pre-post	STN	57 (7,8)	—	10,9 (2,2)	—	3	2,7 (±0,5)	68,7 (±13,9)	38,1 (±17,1)	Quadripolar
Le Jeune et al. [55]	13	France	Pre-post + control	STN	57 (7,8)	—	10,9 (2,2)	—	—	64,6	R 135,3 Hz, L 136,5 Hz	Right 2,3 V y, left 2,4 V	Quadripolar
Castelli, (2010)	27	Italy	Pre-post	STN	60,6 (6,7)	8 (4,1)	15,3 (5,1)	—	1	—	—	—	—
Smeding et al. (2005)	20	Netherlands	Pre-post + control	STN + GP	59,2 (8,6)	10,7 (1,9)	12 (3–50)	2,5 (1,0–5,0)	6, 12	—	—	—	—
Rothlind (2015)	164	USA	Pre-post + control	STN + GPi	62,3 (8,9)	14,8 (3)	12,8 (5,5)	3,3 (0,9)	6	—	—	—	Bilateral
Houvenaghel (2015)	26	France	Pre-post + control	STN	55,8 (6,2)	10,1 (2,4)	11,7 (4)	1,8 (0,8)	3	—	—	—	Bilateral

Pan Test Forward													
Takehiko-Yamanaka (2012)	30	Japan	Pre-post	STN	61,1 (9,1)	12,5 (4,5)	11,5 (5,7)	—	1, 12	90 *μ*s	130 Hz	2,4 V	Monopolar

Corsi Span Backward													
Smeding, (2005)	20	Netherlands	Pre-post + control	STN	59,2 (8,6)	10,7 (1,9)	12 (3–50)	2,5 (1,0–5,0)	6, 12	—	—	—	—
Fasano (2010)	20	Italy	Pre-post	STN	56,9 (7,2)	—	13,7 (4,8)	3	5, 8	60 *μ*s	130 Hz	—	—
Castelli et al. [54]	19	Italy	Pre-post	STN	62,1 (4,2)	—	14,7 (5)	—	17	Right 61,6 (6,9), left 61,6 (6,9)		right 3,2 (0,4), left 3,2 (0,5)	Monopolar bilateral
Castelli, (2010)	27	Italy	Pre-post	STN	60,6 (6,7)	8 (4,1)	15,3 (5,1)	—	1	—	—	—	—

Backward digits													
Takehiko-Yamanaka (2012)	30	Japan	Pre-post	STN	61,1 (9,1)	12,5 (4,5)	11,5 (5,7)	—	1, 12	90 *μ*s	130 Hz	2,4 V	Monopolar
Daniels (2010)	60	Germany	Pre-post + control	STN	60,2 (7,9)	—	13,8 (6,3)	2,29 (0,72)	—	60 *μ*s	130 Hz	Adjusted for each one	Bilateral
Fasano (2010)	20	Italy	Pre-post	STN	56,9 (7,2)	—	13,7 (4,8)	3	5, 8	60 *μ*s	130 Hz	—	—
Fraraccio, (2008)	15	Canada	On-off	STN	58,1 (7,46)	11,3 (3,97)	13,6 (4,39)	—	19	Left: 94,0, right: 94,0	Left: 185 Hz, right: 185 Hz	Left: 2,8, right: 2,8	Quadripolar
Witt et al. [56]	60	Germany	Pre-post + control	STN	60,2 (7,9)	—	13,8 (6,3)	3,62 (0,85)	6	—	—	—	—
Rothlind, et al. (2007)	29	USA	On-off	STN + GP	61,4 (10,11)	15,2 (3,21)	12,9 (4,3)	3,3 (0,45)	—	—	—	—	—
Zangaglia (2009)	32	Italy	Pre-post + control	STN	58,84 (7,70)	7,31 (3,21)	11,84 (5,07)	2,34 (0,43)	1, 6, 12, 24, 36				Semimicroelectrode
Rothlind (2015)	164	USA	Pre-post + control	STN + GPi	62,3 (8,9)	14,8 (3)	12,8 (5,5)	3,3 (0,9)	6	—	—	—	Bilateral
Tang (2015)	27	China	Pre-post	STN					6, 12	—	—	—	Bilateral

Trail Making Test (TMT-A)													
Takehiko-Yamanaka (2012)	30	Japan	Pre-post	STN	61,1 (9,1)	12,5 (4,5)	11,5 (5,7)	—	1, 12	90 *μ*s	130 Hz	2,4 V	Monopolar
Smeding, (2005)	20	Netherlands	Pre-post + Control	STN + GP	59,2 (8,6)	10,7 (1,9)	12 (3–50)	2,5 (1,0–5,0)	6, 12	—	—	—	—
Williams (2011)	19	USA	Post	STN	62,1 (10,3)	13,6 (1,71)	10,1 (6,24)	1,5–3,0	24	—	—	—	—

Stroop													
Williams (2011)	19	USA	Post	STN	62,1 (10,3)	13,6 (1,71)	10,1 (6,24)	1,5–3,0	24	—	—	—	—
Daniels (2010)	60	Germany	Pre-post + control	STN	60,2 (7,9)	—	13,8 (6,3)	2,29 (0,72)	—	60 *μ*s	130 Hz	Adjusted for each one	Bilateral
Smeding, (2005)	20	Netherlands	Pre-post + control	STN + GP	59,2 (8,6)	10,7 (1,9)	12 (3–50)	2,5 (1,0–5,0)	6, 1	—	—	—	—
Moreines, (2014)	17		Pre-post	OTH	1		2		—	91 *μ*s	130 Hz	—	—
Fraraccio, (2008)	15	Canada	On-off	STN	58,1 (7,46)	11,3 (3,97)	13,6 (4,39)	—	19	Left: 94,0, right: 94,0	Left: 185 Hz, right: 185 Hz	Left: 2,8, right: 2,8	Quadripolar
Le Jeune et al. [55]	13	France	Pre-post + Control	STN	57 (7,8)	—	10,9 (2,2)	—	—	64,6	R 135,3 Hz, L 136,5 Hz	Right 2,3 V y, left 2,4 V	Quadripolar
Rothlind, (2007)	29	USA	On-off	STN + GP	61,4 (10,11)	15,2 (3,21)	12,9 (4,3)	3,3 (0,45)	—	—	—	—	—
Le juene, (2010)	13	France	Pre-post	STN	57 (7,8)	—	10,9 (2,2)	—	3	2,7 (±0,5)	68,7 (±13,9)	38,1 (±17,1)	Quadripolar
Witt et al. [56]	60	Germany	Pre-post + Control	STN	60,2 (7,9)	—	13,8 (6,3)	3,62 (0,85)	6	—	—	—	—
Rothlind (2015)	164	USA	Pre-post + control	STN + GPi	62,3 (8,9)	14,8 (3)	12,8 (5,5)	3,3 (0,9)	6	—	—	—	Bilateral
Houvenaghel (2015)	26	France	Pre-post + control	STN	55,8 (6,2)	10,1 (2,4)	11,7 (4)	1,8 (0,8)	3	—	—	—	Bilateral
Tramontana (2015)	30	USA	Pre-post + control	STN + medicam	60 (7)	—	2,2 (1,4)	2	6, 12	—	—	—	Bilateral

Planification													
Zangaglia (2009)	65	Italy	Pre-post + control	STN	58,84 (7,70)	7,31 (3,21)	11,84 (5,07)	2,34 (0,43)	1, 6, 12, 24, 36				Semimicroelectrode
Castelli, (2010)	27	Italy	Pre-post	STN	60,6 (6,7)	8 (4,1)	15,3 (5,1)	—	1	—	—	—	—
Fasano (2010)	20	Italy	Pre-post	STN	56,9 (7,2)	—	13,7 (4,8)	3	5, 8	60 *μ*s	130 Hz	—	—
Castelli et al. [54]	19	Italy	Pre-post	STN	62,1 (4,2)	—	14,7 (5)	—	17	Right 61,6 (6,9), left 61,6 (6,9)		Right 3,2 (0,4), left 3,2 (0,5)	Monopolar bilateral

**Table 2:** 

Neuropsychological test	*k*	*N*	Age	Years PD	DBS	Heterogeneity		
						*Q*	*p*(*Q*)	*I* ^2^
Verbal fluency-semantic	4	141	60,56	12,96	STN			
Verbal fluency-Phonetic	7	178	60,21	13,51	STN	19,769	0,032	49,41
WSCT	2	51	60,47	10,97	STN			
WSCT-Nelson	5	92	58,72	13,1	STN	34,759	0,021	42,46
Trail Making Test-B	8	161	60,91	12,45	STN	5,26	0,511	0,000
Corsi Span Backward	4	86	59,86	14,56	STN			
Digit Span Test	7	246	59,22	13.06	STN-GPi	3,088	0,686	0,000
Trail Making Test-A	3	69	61,6	10,8	STN	0,581	0,748	0,000
Stroop	9	246	65,2	12,18	STN-Cingulate (1)-GPi (1)	102,7	0,001	77,6
Planning	4	98	59,61	13.85	STN			

Note: *k*, number of studies; *N*, number of patients, DBS (deep brain stimulation); *Q*, heterogeneity intradomain; *p*(*Q*)  *p* value of *Q* statistic; *I*^2^, percent of heterogeneity from difference.
