# Corrigendum to “Meta-Analysis of the Relationship between Deep Brain Stimulation in Patients with Parkinson's Disease and Performance in Evaluation Tests for Executive Brain Functions”

**DOI:** 10.1155/2018/9815065

**Published:** 2018-06-28

**Authors:** A. M. Martínez-Martínez, O. M. Aguilar, C. A. Acevedo-Triana

**Affiliations:** ^1^Department of Psychology, Pontificia Universidad Javeriana, Bogotá, Colombia; ^2^Department of Brain Repair and Rehabilitation, University College London, London, UK

In the article titled “Meta-Analysis of the Relationship between Deep Brain Stimulation in Patients with Parkinson's Disease and Performance in Evaluation Tests for Executive Brain Functions” [1], the first affiliation should have been linked to the second author. The revised authors' list and affiliations are shown above.
